# Supplementary material for: Development and Validation of the Patient-Centered Communication Competency Scale for Dental Hygienists
Source: Healthcare (Basel). 2025 May 24;13(11):1241. doi: 10.3390/healthcare13111241 (PMC12155094; doi:10.3390/healthcare13111241)
Supplement: Supplementary file 1 [file healthcare-13-01241-s001.zip › Table S1.pdf]

Table S1

Table S1. Preliminary questions for developing a PCCS scale for dental hygienists

No	Items	1	2	3	4	5
1	I create a comfortable atmosphere when starting communication with the patient.					
2	I greet the patient politely and respectfully upon first meeting.					
3	I clearly state the purpose of each stage of the interview process (e.g., medical history, dental history, chief complaint).					
4	I use words that express respect and interest (e.g., "Are you feeling nervous?").					
5	I use open-ended questions to understand the patient's problem or reason for visiting.					
6	I maintain eye contact with the patient during communication.					
7	I check if the patient has any questions or concerns during the communication process.					
8	I involve the patient in the treatment process and express a sense of partnership (e.g., oral care).					
9	Mirror the patient's words or actions to demonstrate empathy					
10	I do not provide reassurance in a merely formal way when the patient expresses fear about treatment.					
11	Communicate in a structured and proficient manner					
12	I appropriately transition from open-ended to closed-ended questions.					
13	Repeat questions to clarify implied content and emotions					
14	I carefully observe the patient's non-verbal communication (e.g., anxious expressions, nervous hand gestures).					
15	Summarize key points throughout the conversation					
16	Clearly signal transitions between topics or situations.					
17	I use closed-ended questions ("Yes" or "No") to clarify specific information.					
18	Redirect off-topic discussions back to the main topic					
19	Even if I have doubts about what the patient is saying, I avoid immediate objections and instead ask additional questions for clarification.					
20	Clarify expectations regarding diagnosis, treatment, prognosis					
21	I treat the patient with dignity and respect.					
22	I avoid using medical jargon and technical terms as much as possible.					
23	When I must use medical or technical terms, I make sure to explain them clearly.					
24	When conveying treatment-related information, I use drawings or written explanations to aid the patient's understanding.					
25	I encourage the patient to express their emotions.					
26	I listen to the patient without bias and respect their opinions.					
27	I regulate my emotions while communicating.					
28	I identify the patient's beliefs and areas of interest through conversation.					

29	I present information at a pace that the patient can easily understand, breaking it into small parts.					
30	I assess the patient's level of understanding by observing non-verbal cues or directly asking them.					
31	When examining the intraoral C.C. area, I consider the patient's emotional state.					
32	Before checking the intraoral C.C. area, I seek the patient's consent.					
33	During the intraoral C.C. examination, I actively listen to the patient's opinions.					
34	I conduct the intraoral C.C. examination gently, considering the patient's discomfort.					
35	Conclude communication with gratitude for cooperation					
36	Ask if the patient has any additional questions or concerns					
37	At the end of the communication process, I summarize the overall information so that the patient can easily remember it.					
38	Even when documenting the conversation, I show continuous interest in the patient.					

1= Strongly Disagree; 2= Disagree; 3 = Neutral; 4 = Agree; 5 = Strongly Agree
