# Supplementary material for: Development and Validation of the Patient-Centered Communication Competency Scale for Dental Hygienists
Source: Healthcare (Basel). 2025 May 24;13(11):1241. doi: 10.3390/healthcare13111241 (PMC12155094; doi:10.3390/healthcare13111241)
Supplement: Supplementary file 1 [file healthcare-13-01241-s001.zip › Table S2.pdf]

Table S2

Table S2. Final PCCS scale for dental hygienists.

Factors	Items	1	2	3	4	5
Respect	1. Encourage patients to express their emotions freely.<br>환자가 감정을 편안하게 표현할 수 있도록 격려한다.					
	2. Present information by topic at a pace the patient can follow.<br>환자가 잘 이해할 수 있는 속도로, 정보는 주제별 제시한다.					
	3. Mirror the patient's words or actions to demonstrate empathy.<br>환자와 같은 동작이나 말을 반복하여 상대방의 감정에 공감하고 있음을 표현한다.					
	4. Repeat questions to clarify implied content and emotions.<br>환자의 말에 내포 되어있는 내용과 감정을 파악하고자 재질문을 시도한다.					
Assertiveness	5. Communicate in a structured and proficient manner.<br>환자와의 의사소통 과정은 체계적으로 능숙하게 진행한다.					
	6. Summarize key points throughout the conversation.<br>환자와 의사소통 과정에서 대화 내용을 요약해 주며 진행한다.					
	7. Clearly signal transitions between topics or situations.<br>한 주제에서 다른 주제 혹은 다른 상황으로 (예: C.C를 구두로 확인하다가, 구강 내에서 확인시) 전환할 때 이를 명확히 밝힌다.					
	8. Redirect off-topic discussions back to the main topic.<br>환자가 주제와는 다른 방향의 이야기를 할 때 다시 주제로 돌아올 수 있도록 적절한 끊어내기를 한다.					
Empathy	9. Clarify expectations regarding diagnosis, treatment, prognosis.<br>환자가 진단이나, 치료 과정, 치료 예후에 대한 기대가 무엇인지 확인한다.					
	10. Conclude communication with gratitude for cooperation.<br>의사소통과정 마무리 단계에서 협조에 대한 감사 인사를 한다.					
	11. Ask if the patient has any additional questions or concerns.<br>의사소통과정에서 환자에게 다른 질문이나 걱정되는 부분이 있는지 확인한다.					

1= Strongly Disagree; 2= Disagree; 3 = Neutral; 4 = Agree; 5 = Strongly Agree
