# Supplementary material for: Development and Validation of the Patient-Centered Communication Competency Scale for Dental Hygienists
Source: Healthcare (Basel). 2025 May 24;13(11):1241. doi: 10.3390/healthcare13111241 (PMC12155094; doi:10.3390/healthcare13111241)
Supplement: Supplementary file 1 [file healthcare-13-01241-s001.zip › Table S3.pdf]

Table S3. Differences in PCCS by general characteristics

Variables	Categories	n (%)	PCCS Mean ± SD	p-value
Gender	Male	38 (9.5)	45.2 ± 5.67	0.043
	Female	362 (90.5)	43.2 ± 5.70	
Age (yr)	≤ 29	147 (36.8)	43.6 ± 5.52	0.001
	30~39	212 (53.0)	43.0 ± 5.85	
	≥ 40	41 (10.3)	44.4 ± 5.70	
Education	College	222 (55.5)	43.8 ± 5.89	0.001
	University	132 (33.0)	43.3 ± 5.24	
	≥ Master	46 (11.5)	46.3 ± 5.42	
Work place	Dental clinic	300 (75.0)	43.2 ± 5.56	0.293
	Dental hospital	84 (21.0)	44.2 ± 6.34	
	Tertiary hospital	13 (3.3)	41.3 ± 4.39	
	Etc	. (0.8)	44.7 ± 7.64	
Work experience (yr)	≤ 3	70 (17.5)	44.1 ± 5.12	0.416
	≥ 3 ~ ≤ 7	140 (35.0)	43.2 ± 6.18	
	≥ 7	178 (44.5)	43.2 ± 5.51	
Current posision	Clinical dental hygienist	257 (64.3)	43.2 ± 5.61	0.291
	Team leader	53 (13.3)	43.5 ± 6.36	
	Counseling manager	68 (17.0)	43.5 ± 5.52	
	Etc.	22 (5.5)	45.5 ±1.24	
